# Marriage, mental health and the Indian legislation

**DOI:** 10.4103/0019-5545.46067

**Published:** 2005

**Authors:** S. Nambi

**Affiliations:** * Additional Professor of Psychiatry, Madras Medical College

Respected Chairperson, Esteemed Fellows and Members of the Indian Psychiatric Society (IPS), distinguished guests, ladies and gentlemen, I consider this occasion as a great honour bestowed upon me to reach the highest office of our society. I am grateful to the fellows and members of the IPS for giving me the opportunity. After serving this organization in various capacities, being elected as the President and delivering this Presidential address today is a milestone in the growth of my emotional attachment to the IPS.

Marriage has been, since ancient times, one of the most important social institutions—perhaps the greatest and most important of all institutions in human society. It has always existed in one form or another in every culture, ensuring social sanction to a physical union between man and woman and laying the foundation for building up of the family—the basic unit of society.

Sociologists have offered several definitions of marriage:

1. ‘Marriage consists of the rules and regulations which define the rights, duties and privileges of the husband and wife.’—George A. Lundberg
2. ‘Marriage is more or less a durable connection between male and female, lasting beyond the mere act of propagation till after the birth of the offspring.’—Western Marck
3. ‘Marriage is an contractual agreement which formalizes and stabilizes the social relationship which comprise the family.’

Marriage is a socially supported union between individuals in what is intended to be a stable, enduring relationship. It is the basis for the family and the institution, defined by six social functions: regulation of sexual behaviour, reproduction, nurturance and protection of children, socialization, production, consumption, and the passing on of ascribed statuses such as race. Marriage and the family rest on many beliefs, the most important of which is kinship.

In the *shastras* marriage was viewed as a sacrament. The relationship of husband and wife, once established through proper customs and rituals, was believed to be irrevocable. In Hindu marriage, custom is sacrosanct, which is why a marriage ceremony is said to be complete only when the customary rites and rituals are fully performed. Of course, customs vary from place to place and society to society.

Since Hindu marriage is regarded as a means to establish relations between two families, utmost care is taken to ensure maximum cultural compatibility between the two. In Hindu ‘exogamy’, a person has to marry firstly outside his or her own *gotra* and secondly, avoid marrying a person who is a *sapinda* (*sapinda* means ‘of the same body’). The former is called *gotra* exogamy, and the latter is called *sapinda* exogamy. In the Hindu tradition, the observance of custom is supreme and, if custom prevails, marriages between *sapindas* or within the prohibited degrees of relationship are also permissible. For instance, in southern India, marriage between a maternal uncle and niece is valid, whereas in northern India, it is unthinkable due to the absence of such a custom.

Other religions in India include Buddhism, Islam, Sikhism, Christianity and Jainism, which have their own variations of marriage customs. Among Muslim communities, the joint affirmation of the *nikah-nama* (or the marriage contract) is of prime importance. Most Muslims prefer marriage among relations; first cousins are considered most suitable, failing which alliances are sought among families with earlier matrimonial links. This is done to prevent the entry of foreign blood within the family and to retain the property inherited by a young couple.

The *nikah* or marriage service takes place at an hour that is ordained auspicious. The *qazi* or law officer presides over the ceremony, appointing two men as witnesses on the groom’s side, to receive orders for the *nikah* from the bride's family. The amount of *mehr* (a compulsory amount of money given to the bride's family by the groom's family) is negotiated by the elders of the two families.

In Tamil Nadu, self-respect marriage has been introduced by the social reformer Periyar E.V. Ramasamy. The underlying principles of a self-respect marriage are: Marriage is a personal contract between a man and a woman; nothing is sacred, sanctified, religious or divine in the union of man and woman. A contract may be withdrawn by either party or mutually. If marriage is contractual, devoid of religious sanctity, it ceases to be a necessary condition of social life. The male or female has the choice to marry, break away, or not marry at all. In a self-respect marriage, a contract upholds equality of sex, by implication it means that chastity is not a unisexual virtue, but is common to both sexes. Since a contract is breakable, once it is broken, the man and woman may have any other partner by remarriage. Periyar also advises the couple to wait for five years before deciding on pregnancy, and to confine themselves to one or two children, irrespective of its sex and to forget about children if they do not conceive. The woman is not a childbearing machine, says Periyar. Pregnancy is a biological burden, which is an obstacle to a woman's freedom.

There are a variety of marital patterns such as (i) monogamy, (ii) bigamy, (iii) polygamy, (iv) stable marital arrangements/companionship, and (v) same-sex marriage. In most cultures monogamy is ideal.

## CONSANGUINEOUS MARRIAGES AND SAME-SEX MARRIAGES

Many workers in South India have reported a high prevalence of consanguineous marriages. Consanguineous marriages are still common in the states of Tamil Nadu, Andhra Pradesh and Karnataka. There is a strong association between consanguineous marriages and mental disorders.

A study of 147 patients at the Institute of Mental Health, Chennai, suffering from major mental disorders, found that consanguineous parents constituted 36%, and most of them were of second-degree consanguinity. A large series of 86 cases of primary microcephaly showed that in 70% of cases the marriages were consanguineous. A study of 100 mentally retarded children and adolescents attending the outpatient department of the Institute of Mental Health, Chennai, showed that in 28% of cases the marriages were consanguineous, and most of them were of second-degree consanguinity.

Same-sex marriage has been much talked about in recent times. Many countries abhor the basic concept of same-sex marriage; some countries have legalized it. Whether or not same-sex union should be recognized has been discussed in many state legislatures in the West. In India, homosexuality and lesbianism are not only considered taboo, but also an offence. That does not mean that this practice is uncommon here. In India, there are organizations and support groups for the practice of homosexuality and lesbianism. One such organization is Sangini, a social and emotional support group for women attracted towards women. It is a support group for lesbian and bisexual women in India. It should be noted that gay and lesbian psychiatry is one of the specialty sections of the American Psychiatric Association.

Among same-sex common law couples, male couples outnumber female ones. In the United States of America, same-sex couples represented about 1% of all couples in 2000. In New Zealand, the proportion of same-sex couples increased from 0.4% in 1996 to 0.6% in 2001. In the 2001 Canada Census, same-sex couples represented 0.5% of all couples.

‘Gay marriage is good for mental health,’ is what the American Psychological Association said at its annual conference last summer, when it passed a policy statement that same-sex couples should have the right to marry.

Marriage, variously defined as an institution, the bedrock of procreation and family life, a gamble, a life sentence or a heaven-programmed union between two people, is an ideal medium to gauge value shifts in society. Arranged marriages, not surprisingly, remain the most preferred option for most couples, but not arranged in the traditional sense. They are becoming flexible, adaptable, open-minded affairs, based on contemporary, practical and realistic factors. Match-making has come a long way from being the emotionally and socially loaded event ‘fixed’ by the aunt or the family *pundit*. It is now a modern industry chalked out on a bigger canvas with small middlemen playing a key role. Many marriages are fixed via matrimonial columns and matrimonial.coms.

## REFORMS IN HINDU MARRIAGE CUSTOMS

The British succeeded in passing regulation XVII in 1829, which declared *sati* illegal and punishable by courts. The position of women in the nineteenth century was changing, thanks to the endeavours of social reformers like Raja Ram Mohun Roy and Ishwar Chandra Vidyasagar, and later in the twentieth century, in South India by social reformers such as E.V. Ramasamy Periyar. In fact, one of the distinguishing characteristics of modern society is the heavy reliance on law to bring about social change. In India, the first movement for women's marital rights centred on the three major problems of child marriage, enforced widowhood and property rights for women. The traditional concept of marriage has greatly changed now and Hindu marriage today has assumed more or less the nature of a contract for the mutual benefit of the parties concerned, duly aided by various legal provisions and reforms.

## THE CONFLICT PERSPECTIVE OF MARRIAGE (WITH A SPECIAL EMPHASIS ON DIVORCE)

The increasing acceptance of divorce has dramatically altered the marriage situation. While couples still marry at the same constant rate, more than half of all couples married in the USA are now divorced. In India, even though the rate of divorce is not alarming, it is rapidly increasing—presently it is 5%–7%. The vast majority of those divorced remarry, and the second marriage tends to last for the remainder of their lives. There are many in our society who believe that easy marriage and divorce cheapen the institution of marriage and threaten the structure of the family. This may or may not be true, but either way, laws in reality have very little impact on the rates of marriage and divorce. Laws are a reflection of people's needs, they make it easier for people to live with each other and try to ensure that everyone gets fair treatment. Society cannot dictate social and moral behaviour through its laws. If laws do not fit, people will tend to disregard them. This is why so many people choose to live together in the West instead of marrying, as marriage does not fit their situation. Perhaps we would do better to make marriage fit the people, rather than trying to make people fit the institution.

### Divorce

Is it a personal tragedy, a family embarrassment, or a social problem? Or, is it a new lease of life, a chance to start all over again, a chance to make something positive out of something that has turned negative? This is a matter of perspective, depending on how deeply one believes in the ‘till death do us part’ clause of the marriage contract. After months and even years of living with many differences and experiencing fights, frustrations, hurt and anger, a couple may seek divorce as the only viable alternative. At this point, marriage or relationship counselling may provide the couple with new skills in communicating and understanding each other.

Divorce is the emphasis on choice carried to its final limits as far as relationships go. People marry to prove their sense of commitment and to find security. In marriage, however, many find that commitment is situational and security is a figment of the imagination. The concept of divorce prevention revolves around making a mature choice of partner in the first place, making supreme efforts to grow at a parallel pace and continuing to foster the love relationship with care, respect and, above all, good communication.

Family disorganization occurs when statuses are not occupied or roles are not performed. Divorce, death and violence—themselves caused by social and ecological factors—are major causes of family disorganization. The greatest havoc wrought by divorce is the disruption of family life. Divorce in some cultures is no longer viewed as a last resort, but rather as an everyday occurrence. But where marriage is accepted as a sacrament, divorce is bound to be a sacrilege. There are many factors that are responsible for divorce. One of the most important causes has been found to be social change. The process of social change sets into motion a series of changes—in values, in customs, in ways of living, in roles of different people. Technological changes have led to urbanization. Urban society is highly heterogeneous as well as individualistic. The urban attitude is one of non-interference in the affairs of other people. Thus, the social life of urban people also exposes them to a variety of situations that can retract from the bond of attachment to the family. These situations therefore make divorce much easier.

The status of women in the family and society is another factor to be considered. The modern woman, because of her opportunities for education, training and employment, and creative activity, has developed into a self-dependent and self-confident individual. This can lead to difficulties in adjustment in marriages, especially for women who have lived an independent and creative life before marriage. The scope for material prosperity has shifted social values from the spiritual and moral to the material. This change in values has encroached into the realm of marriage too. The wave of progressive liberalism and individualism has made insipid and outmoded the feelings of faithfulness and loyalty. The mass media has played an important role in changing such values. The influence of cinema, television and other media on the younger generation indirectly affects divorce rates. Besides social and cultural differences between the partners, infertility, and the social stigma attached to it, is also a cause for divorce.

## MARRIAGE AND MENTAL HEALTH PROBLEMS

Marriage may be stressful for vulnerable people, which may lead to the development of mental health problems. The interplay between marriage and mental health problems has been dealt with in detail by Indian and international authors. Major mental health disorders may be the cause or effect of marital disharmony. Divorce-seeking couples have a high psychiatric morbidity in comparison to well-adjusted couples with more neurotic traits. The personality factors of divorce-seeking couples also differ from those of couples in stable marriages.

Studies consistently show greater distress among widowed/separated/divorced men and women. Greater distress is seen among married women as compared to married men, and greater distress in single women as compared to single men.

Community surveys in the West showed that women in all categories of marital status were more symptomatic than men in the same categories. After reviewing the epidemio-logical data, linking marital status with illness, it was seen that the ‘effects of gender and marital status vary within ethnicity and psychological disorder’. An ICMR and DST study (1987) on severe mental distress found the highest common distress among housewives in both their urban as well as rural samples. All workers opine that those who were ever married, that is, married/widowed/widower or separated, suffered more than those who never married.

Though some authors feel that in terms of protection, marriage acts as a sort of insurance against psychological breakdown, it need not always be true, because marriage demands a sustained level of adaptation from both partners. The birth of a child, an abortion or miscarriage, economic stress, migration, episodes of illness, major career changes and any situation that involves a significant change in marital role can precipitate stressful periods in a relationship. Illness in a child exerts the greatest strain on a marriage; complaints of lifelong anorgasmia or impotence by marital partners usually indicate intrapsychic problems. Other problems that may induce a marital crisis also trigger off psychological disturbances. They include the discovery of an extramarital affair, onset of serious illness, announcement of intent to divorce, or problems with children or work, one or both members of the couple may be in therapy or may be psychiatrically ill, and one spouse may be seeking hospitalization for the other.

Women in India are less likely to receive mental health care because mental illness in the family, especially in a woman, is itself stigmatizing and an occasion for ridicule. Married mentally ill women are more likely to be sent back to their natal homes, abandoned, deserted or divorced. Clinical experience shows that the responsibility of care for the mentally ill woman is often left to her own family, than to her husband or his family.

### Schizophrenia and marriage

There is evidence that marital status is significantly associated with first admission rates, age of onset, course and outcome of schizophrenia. Single males appear to be overrepresented in schizophrenia samples, including epidemiological studies such as the WHO's 10-country study.^1^ Since both overt schizophrenia and pre-schizophrenic impairments reduce the chances of marriage, married schizophrenics may represent a select group with a milder form of the disease. Alternatively, marriage itself (or living with a partner) may delay the onset of schizophrenia or cushion its effect. Neither of these two hypotheses can be definitely rejected on the basis of available descriptive epidemiological data. However, statistical analysis of the WHO data, in which confounding factors such as age, pre-morbid personality traits, and family history were controlled, found that married men experienced a statistically significant delay (1–2 years) in the onset of psychotic symptoms compared with single men.

Several workers examined the relationship between marriage and schizophrenia. Results from several studies have in common a low marital rate for schizophrenic patients compared with controls and other groups of mentally ill patients, a lower rate in women than in men, a poor clinical course and lower socioeconomic conditions among the divorced, and a clear evidence of selection for schizophrenia among those never married. Often, mental health professionals are faced with having to give advice regarding the marriage of a person suffering from schizophrenia. The answers to many of the questions posed by families are unclear, as there are little research data on which to base counselling on this important issue.

In a 10-year follow up study, Thara and Srinivasan^2^ found that ‘marital outcome in Indian patients is good with no significant gender differences’. The high marital rate (about 70% being married before the onset of the illness), presence of children, a shorter duration of illness at inclusion and the presence of auditory hallucinations at intake were all associated with a good marital outcome. Being unemployed, experiencing a drop in socioeconomic level and the presence of flat affect and self-neglect for 10 years were all associated with a poor marital outcome.

For most women in India, marriage is a one-time event in life, which is glorified and sanctified and is associated with much social approval. It is also the ultimate fulfilment for most women. If this is endangered or broken by mental illness such as schizophrenia, the lives of these women are shattered beyond repair. After separation, almost all these women live with their parents, many of whom are aged. Social isolation and stigma is caused by this double disorganization, of chronic illness and a personal tragedy, stigmatized even now by society. It has brought to the fore the plight of women who, in addition to being affected by a serious mental illness, have also been abandoned by their spouses and left to fend for themselves in a world where few options are open to them. The social, psychological and cultural concomitance of being mentally ill and divorced/separated are particularly severe in the Indian culture. In addition to the stress of mental illness, hostility from family members and rejection from society in general, these women are ridiculed and ostracized for their divorced/separated status. Furthermore, for the families (primarily ageing parents), the emotional, financial and physical burden of caring for a severely mentally ill woman is extremely high. This once again emphasizes the need for educating and bringing greater awareness about mental illness among the people. Many relatives view that severe oddity of behaviour is something that would be set right by marriage. To this mistaken belief about marriage belongs the answer to all these ills. In a study of women with schizophrenia and broken marriages, Thara et al.^3^,^4^ found that the stigma of being separated/divorced is often more acutely felt both by families and patients than that of mental illness *per se*. Caregivers of these separated/divorced/deserted women suffer much more than the patients themselves. Feelings of disruption, loss, guilt, frustration, grief, disappointment and fear about the future of their daughter, all make them miserable. A striking finding from Thara's study was the holistic and very negative attitude of the other family members, such as the siblings, who often anticipating their role as future carers, distance themselves from the patients. This was true also of husbands and in-laws, who often feel cheated. In most arranged marriages, the fact of mental illness is often not disclosed or discussed with the family of the spouse. This is largely due to the fear that disclosure will lead to rejection of the woman. After marriage, in the case of an early relapse, an atmosphere of mistrust and suspicion is created in the family of the spouse that augurs poorly for the outcome of the illness. On the other hand, a psychotic episode after childbirth or after several years of marriage is considered more favourably and does not always result in separation/divorce. The children born to these women suffer from considerable neglect, emotionally and financially, which affects their future mental health.

The family is often considered to be a cause for the onset of schizophrenia. The deviant role relationship and disordered communication pave the way for disordered thinking. Two types of abnormal family patterns were reported: (i) *Marital schew:* in which one parent yielded to the other's (usually the mother's) eccentricities, which dominated the families; (ii) *Marital schism:* in which the parents maintained contrary views so that the child had divided loyalties. It was suggested that these abnormalities were the cause rather than the result of schizophrenia.

In a study of 275 patients at the Institute of Mental Health, Chennai mental illness, marital status and gender differences were analysed (Table 1). Table 1 shows that in the outpatient department of a mental hospital (i) persons suffering from schizophrenia outnumber those with bipolar disorder by 10:1; (ii) the majority of those seeking help are male (M:F = 3:2); (iii) nearly 26% of males and 6% of females are single; 30% of males and 23% of females are married; (iv) 10% of this patient population suffering from a major mental disorder are divorced/separated/widowed. Among these, the number of patients with schizophrenia is 13 times more than those with bipolar disorder.

**Table 1:** Mental illness, marital status and gender differences

							Separated					
									
			Single		Married		Official		Unofficial		Widowed	
							
Diagnosis	Male	Female	Male	Female	Male	Female	Male	Female	Male	Female	Male	Female
Schizophrenia (250)	155	95	65	18	86	56	3	8	6	9	2	7
Bipolar disorder (25)	17	8	4	—	7	3	—	—	0	2	—	—
Total 275	172	103	69	18	93	59	3	8	6	11	2	7

### Marriage and depression

There has been considerable interest in the role of marital status as a risk factor for depression. For men, it appears clear that those married have the lowest rate of depression, while separated or divorced men have the highest rate of major depression. In women, the association is slightly less clear, but in the Epidemiologic Catchment Area (ECA) study, the same findings applied to women as well as men.^5^ Under-standing the nature of the association between marital status and rates of depression is more problematic. If personality is a risk factor for depression, then the same traits can interfere with the ability to marry or to stay married. There is little doubt that depression sometimes contributes to marital maladjustments and separation or divorce. Finally, the stress associated with divorce or separation can increase the likelihood of occurrence of an episode of depression.

In the context of marital relationship, previous research has indicated that for men, marriage confers protection against illness, while it appears to be associated with higher rates of depression for women. There has been some evidence that within marriage, the traditional role of the female is limiting, restricting and even boring, which may lead to depression.

Major depressive disorder occurs most often in persons without close interpersonal relationships or in those who are divorced or separated. Bipolar I disorder is more common in divorced and single persons than among married persons. However, this difference may reflect the early onset and the resulting marital discord characteristic of the disorder. Various social and interpersonal problems may also contribute to depressive relapse. Unhappy marriages seem to be important and a particularly relevant feature of marriage is criticism of the patient by the spouse. Women experiencing marital disharmony suffer from feelings of betrayal, humiliation and shame, which may pave way for developing a depressive disorder.

Children separated from their parents as a result of marital problems and divorce do subsequently have increased rates of depression. Children of divorced parents have more psychological problems than children of parents who are not divorced. It is not certain how far these problems precede the divorce and are related to disharmony between the parents or to the behaviour of one or both parents that contributed to the decision of divorce.

### Alcohol and marriage

Excessive drinking is liable to cause profound social disruption, particularly in the family. Marital and family tension is virtually inevitable. The divorce rate among heavy drinkers is high and the wives of such men are likely to be anxious, depressed and socially isolated. The husbands of battered wives frequently drink excessively, and some women admitted to the hospitals because of self-poisoning blame the drinking habit of their husbands. The home atmosphere is often detrimental to the children because of quarrelling and violence. A drunken parent provides a poor role model. Children of heavy drinkers are at risk of developing emotional and behavioural disorders, and of having poor academic performance at school.

Western studies have shown that almost 80% of domestic violence is due to alcoholism. Indian studies also corroborate this. Addiction seems to be the predominant cause in 50%–60% of cases of domestic violence that have been reported. Marital relationships suffer most harshly from the ravages of addiction. It destroys all that is dear to the spouse, including family life, sexual relationship, economic resources, well-being of the children and status within the community. The wife witnesses the husband's addictive behaviour with increasing abhorrence; often she is also subjected to violence. Women affected by addicted husbands suffer from common difficulties and seem to develop similar behavioural patterns. Never married adults are at the most risk for alcohol and drug dependence.

### Marriage and suicide

Compared with the general population, people who have died of suicide are more likely to have been divorced, unemployed or living alone. Social isolation is a common factor among these associations.

Marriages reinforced by children seem to lessen the risk of suicide significantly. The suicide rate is 11 per 100,000 for married persons, double this rate is registered for those never married/single. Previously married persons, however, showed sharply higher rates than those never married, i.e. 24 per 100,000 among widowed and 40 per 100,000 among those who are divorced, with divorced men registering 69 suicides per 100,000 compared to 18 per 100,000 among divorced women.

Suicide research in India shows that one-fourth of the persons committing suicide are unmarried and the suicide rate is highest in the first year of marriage. Marital and family problems, which constitute around 50%, are more important than mere cruelty. The higher rate of married women committing suicide may probably be due to marital disharmony, dowry or ill-treatment by the in-laws.

### Antisocial behaviour and marital disharmony

Two types of behaviours point indirectly to the importance of parents' behaviour and of enduring family relationships as a cause of antisocial personality disorder. First, there is evidence that parental separation usually follows a long period of tension and arguments that could themselves affect the child's development. Second, the behaviour of parents is an important cause of childhood behavioural disorders. For example, the association between separation and antisocial disorder in sons is determined by disharmony in marriage.

### Anxiety disorder and marriage

Batra and Gautam^6^ found a high prevalence of neurotic disorders among divorce-seeking couples. The neurotic problems are encountered either as antecedents or con-sequences of marital disharmony.

A study comparing marital status in obsessive–compulsive disorder (OCD) patients with that in a matched group of patients with major depression found no significant differences between the two groups. A prospective study of 107 subjects with OCD found that being married significantly increased the probability of partial remission, with married persons being more than twice as likely to remit as unmarried ones.

Patients with schizophrenia are more likely to remain single and unmarried than patients in other diagnostic groups. This is particularly true of male patients and can probably be explained by the fact that women tend to be younger than men when first married and are less likely to have experienced an initial psychotic episode. From a cultural perspective, women are less active in initiating relationships, which may account for some differences.

Some researchers found low rates of fertility and reproduction among patients with schizophrenia. This finding can probably be explained by several factors including lack of interest in social relations, general apathy, loss of sex drive, and lack of opportunity for a sexual relationship due to hospitalization and institutionalization. Rates of reproduction in schizophrenic patients have probably increased since deinstitutionalization, although they are likely to remain lower than those found in the general population.

Some couples ask for help with sexual problems, which are the result and not the cause of marital conflicts. The sexual dysfunction may most probably be due to psychological disturbances such as anxiety and depression, or due to abuse of alcohol and drugs apart from other physical illness.

## MARRIAGE, MENTAL ILLNESS AND THE INDIAN LEGISLATION

Every country and every religion has its own personal law. In India, under Article 44 of the Constitution, the state is bound by a constitutional mandate to secularize and homogenize family laws. The enactment of a uniform civil code was a goal to be achieved through a gradual process. The admixture of religion and ethics with legal precepts was naturally congruent. The practice of applying matrimonial law according to religious faith and beliefs has led to the prevalence of diverse matrimonial laws, besides one statutory law. As per Rule no. 3 of Order 32A of the Civil Procedure Code, it is the duty of the court to make efforts for settlement in matters concerning the family. As per Rule no. 4 of the same Order, a person, preferably a woman, who may or may not be related to the parties including a person professionally engaged in promoting the welfare of the family, may be utilized for the purpose of the settlement mentioned above. According to the Family Court Act, 1984, the Family Court was established with the view to promote conciliation in disputes concerning marriage and related matters. The Family Court may utilize the services of medical and welfare experts for conciliation. This Act reads that persons committed to the need to protect and preserve the institution of marriage shall be selected for appointment as judges and that women shall be preferred for such appointments. It is clear that the intention of law-makers is to prevent the fracture of a family. If a hard decision is inevitable it will be on a specific ground.

The following Acts^7^ have a bearing on the legal aspects of marriage:

1. The Special Marriage Act, 1954
2. The Hindu Marriage Act, 1955 with amendment in 1976
3. The Dissolution of Muslim Marriage Act, 1939; The Muslim Women Protection of Rights on Divorce, 1986
4. The Parsi Marriage and Divorce Act, 1936
5. The Christian Marriage Act, 1872
6. The Indian Divorce Act, 1869
7. The Family Courts Act, 1984

A family arises out of marriage. The single most important factor that influences the quality of family life is the quality of the marriage that supports it. Various socioeconomic changes and developments taking place in the society as a result of industrialization and urbanization have shaken the religious and moral foundations of the institution of marriage. Marriage, according to Hindus and Christians, is a sacred, indissoluble permanent union. Law-makers consider family to be a ‘private sphere’ and recognize the need to respect the privacy by minimizing interference with this institution. Till 1955, matrimonial relief was not available to Hindus and scarcely available to Christians.

The matrimonial relief that one can seek includes:

1. Decree for nullity
2. Restitution of conjugal rights
3. Judicial separation
4. Divorce

Divorce or nullity is granted in cases where a socially accepted marriage is not legally accepted as a marriage, e.g. bigamy. There are two questions with reference to the marriage. Is the marriage a valid one? Is it possible for the relationship to continue? The conditions prevailing at the time of marriage decide its validity. An individual who is not capable of comprehending what is happening to him or her cannot give consent for marriage. The individual may not have the capacity for procreation. The relationship between the parties may be one that prohibited by religious codes. Such situations lay open to question the validity of marriage. Nullity of marriage means that the marriage is held null or void. In other words, a valid marriage does not take place at all. Conditions prevailing in the course of marital life determine the continuation of the relationship between the partners. For example, desertion, cruelty, adultery and mental illness may interfere with marital life and it may not be possible for the relationship to continue. Divorce means that the marriage was a valid one, but the relationship cannot be continued. Following the decree of divorce, the individual becomes eligible for remarriage.

### Muslim law of marriage and divorce

Under this section two Acts need to be taken into account:

1. The Dissolution of Muslim Marriage Act VIII of 1839
2. The Muslim Women (Protection of Rights on Divorce) Act, 1986.

A Muslim who is of sound mind and has attained puberty (presumed to have been attained on completion of 13 years, unless the contrary is proved) is qualified to marry. Under the Muslim marriage law, lunatics or persons of unsound mind and minors who have not attained puberty may be validly contracted into marriage by their respective guardians.

According to Muslim law, marriages are of three kinds: (i) *sahih* or valid; (ii) *batil* or void; and (iii) *fasil* or irregular. The Muslim law, unlike the Special Marriage Act, 1954 and the Hindu Marriage Act, 1955, does not classify any marriage as voidable marriage. A *sahih* or valid marriage is one that is contracted by observing all the conditions of marriage. If some of those conditions are violated, the marriage is *batil* or void. Those conditions are: consanguinity, affinity, forterage, marriage by a Muslim woman without divorce by a living husband. If some other conditions are violated the marriage is declared *fasil* or irregular.

Irregular (*fasil*) marriages are: (i) marriage contracted without witnesses; (ii) marriage with a fifth wife when four wives are present; (iii) marriage during the *iddat* period; (iv) marriage prohibited on the grounds of a different religion; (v) marriage prohibited as an unlawful conjunction.

Mutah marriage (*nikah-i-mutah*): A *mutah* marriage is recognized among the Muslims of the Shia sect as a temporary marriage. Its duration can be fixed by an agreement between the two parties. The Sunni sect does not recognize this kind of marriage.

The All India Muslim Personal Law Board (AIMPLB) has urged Muslims to avoid the practice of triple *talaq*, while its executive committee approved the draft of a model ‘*nikah-nama*’ which contains guidelines for married couples. The AIMPLB said that Muslim couples should hesitate in giving *talaq*, particularly the triple *talaq*, which is considered to be a sin.

#### Dissolution of marriage as per the Muslim Law

A Muslim marriage can be dissolved by divorce by the parties without recourse to the court and on certain grounds by recourse to the court.

The law as ordained by the Holy Quran is that *talaq* must be for a reasonable cause and that it must be preceded by an attempt at reconciliation between the husband and the wife by two arbiters, one chosen by the wife from her family and the other by the husband from his family. If their attempts fail then *talaq* may be effective.

#### Dissolution of marriage without recourse to court

*Talaq*: When divorce proceeds from a husband to his wife it is known as divorce by *talaq*. A Muslim husband of sound mind who has attained the age of puberty may divorce his wife whenever he desires without assigning any cause. The dictionary meaning of *talaq* in the language of law is ‘taking off of the marriage tie by appropriate words’.

There are two kinds of *talaq* on the basis of effect:

1. Revocable (*rujaee*)
2. Irrevocable (*bain*)

There are three kinds of *talaq* based on the forms:

1. *Talaq absun*: This is done by a single pronouncement of divorce. If the marriage is not consummated, *talaq* can be pronounced in this manner.
2. *Talaq hasun*: This consists of three pronouncements made after an interval of a month. On the third pronouncement talaq becomes irrevocable.
3. *Talaq-ul-bidat*: Three pronouncements made at shorter intervals or even in quick succession. This mode is considered sinful.

*Talaq* may be through spoken words (oral) or by a written document (*talaq-nama*).

#### Divorce by recourse to court (judicial divorce)

According to the Muslim Marriage Act, 1939, a woman married under the Muslim law shall be entitled to obtain a decree for the dissolution of her marriage (based on mental health issues) on the following grounds:

1. That the husband has been insane for a period of 2 years.
2. That the husband was impotent at the time of marriage and continues to be so. Inability of the husband to consummate the marriage is one pattern of impotence.

### Parsi Law: Parsi Marriage and Divorce Act, 1936, amended in 1988

A Parsi means a Zoroastrian and the Act is meant for this community. The Parsi Law does not prescribe any specific form of marriage; however, one essential ceremony must be performed in all Parsi marriages. This is the Ashirvad ceremony. No Parsi marriage shall be valid unless it is solemnized by the Ashirvad ceremony. Under the Parsi Marriage and Divorce Act, unsoundness of mind is not a ground for annulment of marriage.

#### Grounds for divorce in the Parsi Marriage Act

**Section 32:** Any married person may seek divorce on the following grounds: That the defendant at the time of the marriage was of unsound mind and has been habitually so up to the date of the suit; provided that divorce shall not be granted on this ground, unless the plaintiff (i) was ignorant of the fact at the time of the marriage, and (ii) has filed the suit within three years from the date of the marriage.

**Section 32 (b) (Amendment Act, 1988):** That the defendant has been incurably of unsound mind for a period of two years or upwards immediately preceding the filing of the suit or has been suffering continuously or intermittently from mental disorder of such kind and to such an extent that the plaintiff cannot reasonably be expected to live with the defendant.

‘Mental disorder’ explained in this section is similar to that in the Special Marriage Act and the Hindu Marriage Act.

### Christian law of marriage and divorce and the Indian Divorce Act (The Divorce Act, 1869)

**Dissolution of marriage:** The Indian Divorce Act, 1869; amended in 2001.

On the demand of several Christian organizations, Section X of the IDA was amended by Act No. 51 of 2001. As per this amendment, the grounds for divorce are very much similar to those under the Special Marriage Act and the Hindu Marriage Act.

**Section X (I) (II):** Unsoundness of mind. This is a ground for divorce on two conditions: (i) The unsoundness of mind must be ‘incurable’ and medical evidence is required to prove it. (ii) It must be present for at least two years immediately before the filing of the petition. It is submitted that both the conditions must run together. If the respondent's unsoundness of mind was curable in the beginning, but later on became incurable, the period of two years will be counted from the date when the disease became incurable.

The Christian wife has some exclusive grounds for divorce: **Section X (II):** The three exclusive grounds for divorce that a wife can file are (i) rape, (ii) sodomy, and (iii) bestiality. Dissolution of marriage by mutual consent in now possible for Christians under Section X A of the IDA (Amendment Act), 2001.

**Nullity of marriage:** The grounds for nullity of marriage as per the IDA Section 19: Impotence, lunacy or idiocy are among the five causes for nullity. Under Section 19 (3), it must be established that the respondent was a lunatic or idiot at the time of marriage.

### The Special Marriage Act, 1954

The Special Marriage Act is meant for any person in India and Indian nationals abroad, irrespective of the faith that the individual may profess. A marriage solemnized in any other form can be registered under this Act. The enactment of 1954 has been subsequently amended. The Marriage Laws (Amendment) Act, 1976 has brought about considerable changes in the grounds for divorce, judicial separation and nullity.

#### Conditions of special marriages

The section lays down five conditions of marriage:

1. Monogamy
2. Mental soundness (Clause b)
3. Marital age
4. Relationship (Clause d)
5. Conditions of marriage in J&K (Clause e)

**Mental soundness:** Marriage is tagged with lifelong responsibilities. The Special Marriage Act, therefore, provides that neither party:

- is incapable of giving a valid consent to it as a consequence of unsoundness of mind; or
- though capable of giving valid consent, has been suffering from mental disorder of such a kind or to such an extent as to be unfit for marriage and the procreation of children, or
- has been subject to recurrent attacks of insanity.

The condition of mental soundness for marriage does not mean that the person to be married must possess a high intelligence quotient. It only requires that he should understand the special nature of the relationship that marriage creates.

The present provisions of Section 4(b) are substituted by the Marriage Laws Amendment Act, 1976. The original provision was ‘neither party is an idiot or a lunatic’.

According to the Marriage Laws (Amendment) Act, 1976, recurrent epilepsy was also a disqualification for marriage. Now that has been removed by the Marriage Laws (Amendment) Act (No. 39 of 1999) with effect from December 1999. Marriage in violation of this condition is void u/s 24(I)(1).

## GROUNDS FOR JUDICIAL SEPARATION

Section 23 provides for judicial separation on any of the grounds for divorce specified in Section 27, sub-Sections I and IA.

**Unsound mind:** That the respondent has been incurably of unsound mind or has been suffering continuously or intermittently from mental disorder of such a kind and to such an extent that the petitioner cannot reasonably be expected to live with the respondent.

### Ground for divorce: Section 27(l)

A petition for divorce may be presented to the District Court either by the husband or the wife on the ground that the respondent, as per Clause (e), has been incurably of unsound mind or has been suffering continuously or intermittently from mental disorder of such a kind and to such an extent that the petitioner cannot reasonably be expected to live with the respondent. In this clause;

- The expression of mental disorder means mental illness, arrested or incomplete development of the mind, psychopathic disorder or any other disorder or disability of mind and includes schizophrenia.
- The expression ‘psychopathic disorder’ means a persistent disorder or disability of the mind (whether or not including subnormality of intelligence) which results in abnormally aggressive or seriously irresponsible conduct on the part of the respondent and whether or not it requires or is susceptible to medical treatment.

### Hindu Marriage Act, 1955

This is an Act to amend and codify the law relating to marriage among Hindus. This enactment of 1955 has been subsequently amended eight times from 1956 to 2003. The Marriage Laws (Amendment) Act, 1976 has brought about considerable changes in the original Act. The Hindu Marriage Act applies to Hindus, Buddhists, Jains and Sikhs. The Act of 1955 provided for four types of matrimonial relief: (i) restitution of conjugal rights, (ii) judicial separation, (iii) declaration of nullity and annulment, and (iv) divorce. Divorce was the most radical social reform. The *dharmasashtras* did not recognize divorce. By 1955, marriage came to be accepted as essentially a contract. The issue of divorce then comes as a natural consequence.

Adultery, desertion and cruelty were looked upon as ‘matrimonial offences’ which needed to be punished and divorce was used as an instrument for punishing people guilty of such offence. This was initially called the ‘offence theory’ of divorce. Later, insanity was added as a ground for divorce and came to be regarded as a ‘matrimonial offence’, though insanity or unsoundness of mind is a misfortune, not a cause for guilt. Since diseases like insanity could not be categorized as an offence, the ‘offence theory’ was renamed as the ‘fault theory’. That is, if the respondent had ‘some fault’, which made continuance of cohabitation at most impossible, the petitioner was entitled to divorce.

#### Condition for a Hindu Marriage

**Section 5:** A marriage may be solemnized between any two Hindus, if the following conditions are fulfilled: (i) neither party has a spouse living at the time of marriage; (ii) at the time of marriage neither party (a) is incapable of giving valid consent in consequence of unsoundness of mind, or (b) though capable of giving valid consent, has been suffering from mental disorder of such a kind or to such an extent as to be unfit for marriage and the procreation of children, or (c) has been subject to recurrent attacks of insanity (the words ‘or epilepsy’ were omitted by Act 39 of 1999, Section 2); (iii) the bridegroom has completed 21 years and the bride has completed 18 years at the time of marriage; (iv) the parties are not within the degree of prohibited relationships; (v) the parties are not *sapindas* of each other unless the custom or usage governing each of them permits of a marriage between the two.

#### Section 5, Clause (ii): Mental health

This clause requires that neither party to the marriage must be incapable of giving valid consent because of unsoundness of mind. The expression ‘unsoundness of mind’ has to be understood as lack of a state of mind or capacity to understand one's affairs or marital obligations.

#### Original provision

This clause has been amended by the Marriage Laws (Amendment) Act, 1976. The original provision was, ‘neither party is an idiot or lunatic at the time of marriage’.

The term ‘unsoundness of mind’ in the present provision is more comprehensive. Idiocy and lunacy are types of unsoundness of mind. Idiocy is manifested by indecency of behaviour, dirtiness in habits or by vacancy of aspect or by inability to understand the commonest rules of arithmetic. Unsoundness of mind is depravity or ‘mania’. A person of dull or feeble intellect may not understand the nature of a marital relationship.

Unsoundness of mind is not the same thing as ‘feeble mindedness’ or ‘dullness of intellect’. It is not a disquali-fication for marriage because a person of dull or feeble intellect may understand the nature of a marital relationship. In addition, neither party should be suffering from mental disorder of a type and to an extent as to render the party unfit for marriage and the procreation of children. A party could be said to be unfit for marriage if the party is not able to understand the nature and implication of a marital relationship. A party would be unfit for procreation if the party would not be able to look after or maintain the children from the marriage, or the children would be likely to be suffering from the same mental disorder or defect. The word ‘and’ between the expression ‘unfit for marriage’ and ‘procreation of children’ should be read as ‘and/or’. The court can nullify the marriage if either condition or both the conditions contemplated exist. The word ‘procreation’ includes the capacity to rear children besides the capacity to beget them.

In the further alternative neither party should have been subject to recurrent attacks of insanity meaning ‘subject to an increase of the acuteness or severity of unsoundness of mind recurring periodically in its course’.

A marriage in contravention of this condition is not void but voidable under Section 12, sub-Section (2), Clause (b).

#### Grounds for judicial separation

On reading Section 10 and Section 13 together, relief of judicial separation would be available on the following grounds:

That the respondent has been incurably of unsound mind, or has been suffering continuously or intermittently from mental disorder of such a kind and to such an extent that the petitioner cannot reasonably be expected to live with the Respondent. *See* Section 13(1)(iii).

#### Nullity

Unsoundness of mind or mental disorder or insanity—contravention of conditions specified in Clause (ii) of Section 5—Section 12(1)(b).

A marriage becomes voidable if the respondent was incapable of giving consent as a consequence of unsoundness of mind or, though the respondent was capable of giving valid consent, the respondent was suffering from mental disorder of such a kind or extent as to be unfit for marriage and the procreation of children; or the respondent has been subject to recurrent attacks of insanity. The Supreme Court held in R. Lakshmi Narayan versus Santhi that to brand a wife as unfit for marriage and procreation of children on account of a mental disorder, it needs to be established that the ailment suffered by her is of such a kind or to such an extent that it is impossible for her to lead a normal married life.

The unfitness for marriage and procreation of children contemplated here is one arising from mental disorder only, and not on account of any other disorder. Infertility or sterility as such is not a ground for annulment of marriage under Section 12 or for divorce under Section 13.

In this case, the respondent was at the time of marriage suffering from schizophrenia. This disease of the mind affects normal behaviour. A person having this illness can be termed ‘not sane’. Therefore, it was held that the case was covered by Section 12(1)(b) read with Section 5(ii)(c) of the Act.

**Section 13(1)** Any marriage solemnized, whether before or after the commencement of this Act, may, on a petition presented by either the husband or the wife, be dissolved by a decree of divorce on the ground that the other party:

**Section (iii)** has been incurably of unsound mind, or has been suffering continuously or intermittently from mental disorder of such a kind and to such an extent that the petitioner cannot reasonably be expected to live with the respondent.

- the expression ‘mental disorder’ means mental illness, arrested or incomplete development of mind, psychopathic disorder or any other disorder or disability of mind and includes schizophrenia;
- the expression ‘psychopathic disorder’ means a persistent disorder or disability of mind (whether or not including subnormality of intelligence) which results in abnormally aggressive or seriously irresponsible conduct on the part of the other party, and whether or not it requires or is susceptible to medical treatment;

**Section 13(1)(iii):** Incurably of unsound mind or suffering from mental disorder. The expression ‘incurably’ of unsound mind cannot be so widely interpreted as to cover feeble-minded persons or persons of dull intellect who understand the nature and consequences of the act and are therefore able to control them and their affairs, and their reaction in the normal way (A.S. Mehta versus Vasumathi (A.I.R. 1969) Guj–48; Parvathi Mishra versus Jagadhanantha Mishra (1994) 78 CLT 561. When there was sufficient evidence for the court to conclude that the slight mental disorder of the wife was not of such a kind and to such an extent that the husband could not reasonably be expected to live with her, divorce could not be granted (Rita Roy versus Sitesh Chandra, A.I.R. 1982) CAL 138; 86 CWL 167. Each case of schizophrenia has to be considered on its own merits.

The medical evidence regarding the requisite degree of mental disorder is relevant, though not conclusive (Sharada versus Dharmapaul (2003) 4 SCC 493). In so far as granting the relief of divorce under Section 13(1)(iii) of the Act is concerned, the nature and degree of mental disorder which meets the requirements has been clearly discussed and spelt out in one of the important cases (Ramnarayan Gupta versus Sreemathi Rajeshwari Gupta, Justice Venkatachaliah [Supreme Court 1998]). ‘Each case of mental illness or schizophrenia has to be considered on its own merits.’ Schizophrenia is what schizophrenia does. The judgment is significant because it gives importance to the effects and the impact rather that to the mere labelling of mental illness.

A Division Bench of the Andhra Pradesh High Court held in Hema Reddy versus Rakesh Reddy (2003) that psychological depression by itself is no ground for divorce under the Hindu Law.

### Impotence

Impotence means the incapacity to perform sexual intercourse which is full and natural. Refusal to have sex is different from impotence. Impotence is different from sterility. Consum-mation means full penetration, not attempt to penetrate. Based on impotence at the time of marriage as per Section 19(1) of the IDA and as per Section 30 of the Parsi Marriage Act, and as per Section 24(ii) of the Special Marriage Act and according to Section 12A of the Hindu Marriage Law, the marriage becomes null and void.

According to Section 2(v) of the Dissolution of Marriage Act, one of the grounds for dissolution of marriage is impotence. The institution of suit should be applied for within one year for nullity and after one year for divorce.

### Nagging by spouse: A ground for divorce

In a recent judgment (December 2004), the Supreme Court held that a spouse can seek divorce if he or she is subjected to mental agony and cruelty due to constant nagging by his or her partner. This judgment invoked a lot of debate. Regarding this judgment, a magazine conducted a survey in Tamil Nadu: 56.25% supported the judgment and 37.5% opposed it; 65% of those surveyed accepted that they were nagged by their partners.

**Box 1:** Unsoundness of mind as affecting the capacity to marry

**Hindu Marriage Act, 1955**
*Before 1976*
Neither party is an idiot or a lunatic at the time of marriage.
*After 1976*
A Hindu marriage is voidable if either party:
is incapable of giving valid consent as a consequence of unsoundness of mind, or though capable of giving valid consent has been suffering from mental disorder of such a kind or to such an extent as to be unfit for marriage and the procreation of children has been subject to recurrent attacks of insanity or epilepsy.
**The Special Marriage Act, 1954**
Applicable to persons from any religion undergoing a civil marriage. Has provisions similar to the HMA except that a marriage under the SMA is void.
**Indian Divorce Act, 1869**
A Christian marriage is voidable if either party was a lunatic or idiot.
**Parsi Marriage and Divorce Act, 1936**
Unsoundness of mind is not a ground for annulment.
**Muslim Law**
A person of unsound mind cannot contract a marriage and such a marriage, if contracted, is void. However, if the guardian of the person of unsound mind considers such a marriage to be in his interest and in the interest of society and is willing to take up all the monetary obligations of the marriage, then such a marriage can be performed.
**Christian Law of Marriage and Divorce**
Unsoundness of mind is a ground for divorce on two conditions—that is, it must be incurable and it must be for at least two years immediately before the filing of the petition.

In a compilation of 61 cases of divorce, Dhanda^8^ found that divorce/nullity had been filed on the following facts: Hindu Marriage Act: 49, IDA: 11 and Parsi Act: 1. Out of 34 cases filed for divorce only 10 cases were granted divorce, and out of 30 cases for nullity of marriage, 12 cases were granted the same. In her compilation of 61 cases, Amita Dhanda found that schizophrenia forms nearly one-third of the diagnostic pattern of causes followed by mild mental disorder/insanity/unsoundness of mind. The other categories are mental retardation, epilepsy and manic–depressive psychosis. In the Family Court of Chennai, during the past one year, nearly 5000 cases had been filed for dissolution/nullity. Out of these only 45 cases were filed on the ground of mental illness. Of these, 42 cases were filed under the Hindu Marriage Act and 3 cases under the IDA. This finding indicates that in our culture the basis of mental disorder for filing a divorce suit is not significant.

Mental illness is a question of fact. It has to be proved in court. It is not a matter of interpretation. The law presumes that sanity and insanity have to be proved. The standard of proof is the preponderance of probabilities. It means that the probability of insanity should be more than the improbability of sanity. The court comes to a conclusion on the basis of not only medical evidence, but also other pieces of evidence. It will be of assistance to the court if the psychiatrist adds a description of the observable behaviour in the report. The burden of proving the insanity of the respondent rests on the petitioner. It is the responsibility of the psychiatrist to keep the documents sound. A certificate given by the psychiatrist is only a statement of opinion, and it attains the status of evidence only when its author undergoes cross-examination. The court need not accept the opinion of the psychiatrist and the medical evidence can be rejected.

The law should not discourage persons from seeking treatment for mental disorders, rather it should ‘perform a promotive and facilitative role, it is suggested that an express legislative provision should be incorporated, which states that a past history of mental illness will be no bar to marriage; failure to disclose such past history or the fact of treatment would not amount to suppression of a material fact’.^8^ The psychiatrist must know that the intention of the court is similar to his/hers. Both do not want to promote the incidence of broken homes. The decision of the court either preserves or breaks a family. The psychiatrist must be aware of the legal provisions, in order to meet the legal requirements.

In conclusion, it is important to recognize that although the legislation provides the constitutional and legal perspectives and sets standards for cases being settled through the process of law, the social problems of this nature have to be also worked through and resolved in many other ways. Legislation is necessary, but the promotive and harmony-seeking approaches are equally important, and indeed are more likely to be useful.
